# (*Z*)-1,2:5,6-Di-*O*-isopropyl­idene-α-d-*ribo*-hexofuranos-3-ulose *O*-benzyl­oxime

**DOI:** 10.1107/S1600536809006333

**Published:** 2009-02-28

**Authors:** Anja Burkhardt, Lars Eriksson, Göran Widmalm, Ian Cumpstey

**Affiliations:** a Department of Organic Chemistry, Arrhenius Laboratory, Stockholm University, SE-106 91 Stockholm, Sweden; b Division of Structural Chemistry, Arrhenius Laboratory, Stockholm University, SE-106 91 Stockholm, Sweden

## Abstract

The title compound, C_19_H_25_NO_6_, is a *Z* diastereomer in which the phenyl ring of the 3-benzyl­oxime substituent and the 5,6-*O*-isopropyl­idene acetal are both located on the *Si*-face of the C=N double bond. Inter­molecular C—H⋯O inter­actions result in helical chains along the *b* axis of the monoclinic unit cell.

## Related literature

For background to sugar-based oxime derivatives, see: Tronchet *et al.* (1979 ▶, 1981 ▶, 1989 ▶); Peri *et al.* (2004 ▶). For the synthesis, see: Plenkiewicz *et al.* (1974 ▶); Fernández-González & Alonso (2006 ▶). For ring puckering analysis, see: Cremer & Pople (1975 ▶). For C–H⋯O inter­actions, see: Gatti *et al.* (2002 ▶). For the synthesis of a reactant, see: Shing *et al.* (1996 ▶).
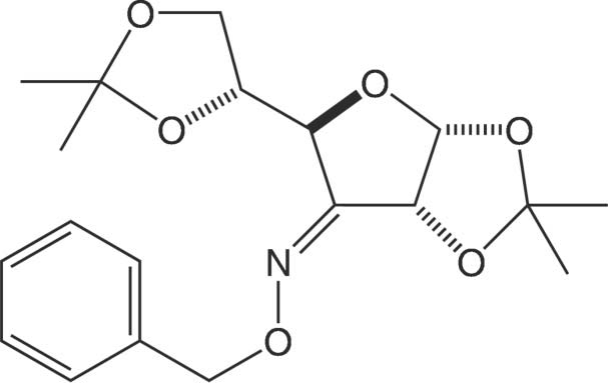


## Experimental

### 

#### Crystal data

C_19_H_25_NO_6_
*M* *_r_* = 363.40
Monoclinic, 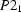

*a* = 11.8012 (12) Å
*b* = 6.0019 (5) Å
*c* = 13.7021 (11) Å
β = 95.122 (11)°
*V* = 966.64 (15) Å^3^
*Z* = 2
Mo *K*α radiation
μ = 0.09 mm^−1^
*T* = 293 K
0.30 × 0.05 × 0.04 mm

#### Data collection

Stoe IPDS diffractometer
Absorption correction: none
9449 measured reflections
2558 independent reflections
2023 reflections with *I* > 2σ(*I*)
*R*_int_ = 0.104

#### Refinement

*R*[*F* ^2^ > 2σ(*F* ^2^)] = 0.052
*wR*(*F* ^2^) = 0.131
*S* = 0.99
2558 reflections
235 parameters
1 restraint
H-atom parameters constrained
Δρ_max_ = 0.31 e Å^−3^
Δρ_min_ = −0.29 e Å^−3^

### 

Data collection: *EXPOSE* in *IPDS Software* (Stoe & Cie, 1997 ▶); cell refinement: *CELL* in *IPDS Software*; data reduction: *INTEGRATE* in *IPDS Software*; program(s) used to solve structure: *SHELXS97* (Sheldrick, 2008 ▶); program(s) used to refine structure: *SHELXL97* (Sheldrick, 2008 ▶); molecular graphics: *XP* (Siemens, 1990 ▶); software used to prepare material for publication: *PLATON* (Spek, 2009 ▶), *XP* and *SHELXL97*.

## Supplementary Material

Crystal structure: contains datablocks I, global. DOI: 10.1107/S1600536809006333/ng2545sup1.cif

Structure factors: contains datablocks I. DOI: 10.1107/S1600536809006333/ng2545Isup2.hkl

Additional supplementary materials:  crystallographic information; 3D view; checkCIF report

## Figures and Tables

**Table 1:** Hydrogen-bond geometry (Å, °)

*D*—H⋯*A*	*D*—H	H⋯*A*	*D*⋯*A*	*D*—H⋯*A*
C5—H5⋯O2^i^	0.98	2.43	3.386 (3)	164
C10—H10*A*⋯O3^ii^	0.97	2.46	3.387 (3)	160

Symmetry codes: (i) 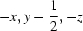; (ii) 
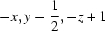
.

**Fig. 1.**
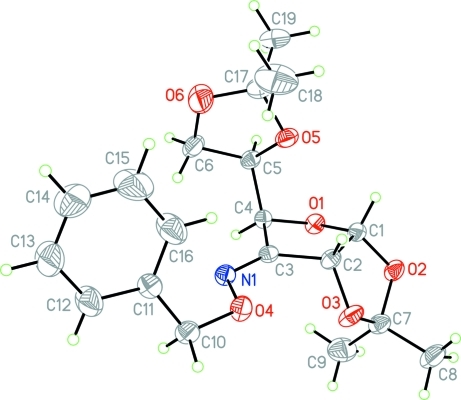
Molecular structure of the title compound (I) with displacement ellipsoids at 30% probability and atom-numbering scheme.

**Fig. 2.**
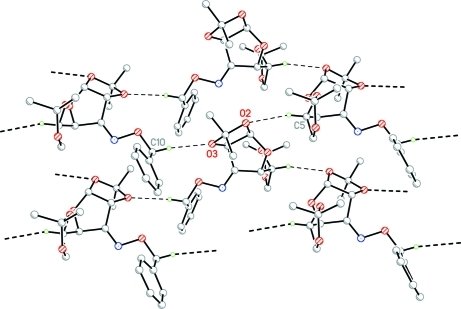
Molecules of (I) linked by C–H···O interactions resulting in chains with twofold screw symmetry along the crystallographic b axis.

## supplementary crystallographic information

### Comment

Oxime derivatives of carbohydrates are important precursors for preparation of monosaccharide building blocks in disaccharide mimic synthesis. (Tronchet *et al.*, 1979, 1981,1989; Peri *et al.*, 2004)

The title compound (I) can be easily synthesized by condensation of the corresponding keto sugar with *O*-benzylhydroxylamine hydrochloride in pyridine in good yield. (Plenkiewicz *et al.*, 1974; Fernández-González & Alonso, 2006) Crystallization from pentane/ethyl acetate gave exclusively the *Z* diastereomer of I. The crystal structure of I is depicted in Fig. 1 and reveals a twist form for the sugar furanose ring (O1, C1, C2, C3, C4) according to the puckering parameters *q*_2_ = 0.257 (2) Å and *Φ*_2_ = 164.8 (5)°. (Cremer & Pople, 1975) This conformation is also observed for the 1,2-*O*-isopropylidene (O2, C1, C2, O3, C7; *q*_2_ = 0.237 (2) Å, *Φ*_2_ = 162.1 (6)°) and the 5,6-*O*-isopropylidene acetal (O5, C5, C6, O6, C17; *q*_2_ = 0.310 (3) Å, *Φ*_2_ = 15.4 (6)°). The phenyl ring of the 3-benzyloxime substituent at C3 and the furanose mean plane (O1, C1 to C4) are twisted to each other by 87.2 (2)° indicated by an N1—O4—C10—C11 torsion angle of -74.9 (3)°. The *Re*-face of the C=N double bond is shielded by a methyl group of the 1,2-*O*-isopropylidene entity, while the *Si*-face is shielded by the benzyl group of the 3-benzyloxime substituent and the 5,6-*O*-isopropylidene protecting group. Assuming that the latter two groups are able to rotate in solution and the 1,2-acetonide is rigid, a nucleophilic attack from the *Si*-face should be preferred.

The donor-acceptor distance between O3 and the benzylic carbon is 3.387 (3) Å and is therefore in the range of C—H···O hydrogen bonds. (Gatti *et al., *2002) Furthermore, H5 is hydrogen bonded to O2 of an adjacent molecule with a donor-acceptor distance of 3.386 (3) Å. These weak intermolecular interactions lead to chains of molecules of I featuring a twofold screw symmetry along the crystallographic *b* axis (see Fig. 2).

### Experimental

All substances were purchased from commercial suppliers and used without further purification. Pyridine was dried over molecular sieves 4 Å. 1,2:5,6-Di-*O*-isopropylidene-α-*D*-*ribo*-hexofuranos-3-ulose was obtained from 1,2:5,6-di-*O*-isopropylidene-α-D-glucose (Shing *et al.*, 1996)

Melting points are given uncorrected and were determined with a Gallenkamp melting point apparatus. Mass spectra were measured on a Bruker Daltonics MicroTOF spectrometer. Optical rotations were determined using a Perkin-Elmer 241 polarimeter with a path length of 1 dm (Concentrations are given in g/100 ml.). ^1^H NMR, ^13^C NMR, ^1^H,^1^H COSY and ^1^H,^13^C HSQC experiments were carried out on a Bruker 400 MHz s pectrometer. Chemical shifts are referenced for ^1^H to CHCl_3_ at 7.26 p.p.m. and for ^13^C to CDCl_3_ at 77.03 p.p.m.. Multiplicities are quoted as singlet (*s*), doublet (*d*), doublet of doublets (dd), doublet of doublet of doublets (ddd) and multiplet (*m*). Coupling constants *J* are determined from zero-filled, resolution enhanced spectra.

(*Z*)-1,2:5,6-di-*O*-isopropylidene-α-*D*-*ribo*-hexofuranos-3- ulose *O*-benzyloxime (I): 1,2:5,6-Di-*O*-isopropylidene-α-*D*-*ribo*-hexofuranos-3-ulose (2.96 g, 11.5 mmol) was dissolved in pyridine (20 ml). After addition of *O*-benzylhydroxylamine hydrochloride (2.75 g, 17.2 mmol) the mixture was stirred at room temperature under nitrogen. After 26 h, TLC (CH_2_Cl_2_/ethyl acetate, 20:1) showed conversion of the starting material (*R*_f_ = 1/5) to a major product (*R*_f_ = 1/2). The mixture was concentrated *in vacuo.* The resulting white solid was dissolved in CH_2_Cl_2_/ethyl acetate. The organic phase was washed three times with water, dried over Na_2_SO_4_, filtered and concentrated *in vacuo*. Recrystallization from pentane/ethyl acetate (2:1) at 246 K gave (*Z*)-1,2:5,6-di-*O*-isopropylidene-α-*D*-*ribo*-hexofuranos-3- ulose *O*-benzyloxime (I) as colourless crystals. After filtration the product was dried *in vacuo* at 298 K. Yield: 3.10 g (74%). Single crystals suitable for X-ray determination were obtained after one week of gas diffusion of pentane into a solution of I (20 mg, 0.06 mmol) in ethyl acetate (0.5 ml). *M*.p. = 402–404 K (pentane/ethyl acetate). [α]_D_^21^ = +201 (c = 1.0 in CHCl_3_). ^1^H NMR (400 MHz, CDCl_3_, 298 K): 1.28, 1.33, 1.43 and 1.49 (4 s, 12H, H methyl), 3.77 (dd, ^2^*J*_66'_ = 8.3 Hz, ^3^*J*_65 _= 7.2 Hz, 1H, H6), 3.84 (dd, ^3^*J*_6'5 _= 6.8 Hz, 1H, H6'), 4.36 (ddd, ^3^*J*_54_ = 2.6 Hz, 1H, H5), 4.84 (dd, ^4^*J*_42 _= 1.3 Hz, 1H, H4), 5.16 (dd, 1H, H2), 5.20 (2*H*, H10 and H10'), 5.95 (d, ^3^*J*_12_ = 4.4 Hz, 1H, H1), 7.29–7.36 (m, 5H, H Ph) p.p.m.. ^13^C NMR (100 MHz, CDCl_3_, 298 K): 25.5, 26.0, 27.3 and 27.4 (4 × q, C8, C9, C18 and C19), 64.2 (t, C6), 74.8 (d, C2), 76.9 (× 2) and 77.0 (2 × d and 1 × t, C4, C5 and C10), 104.8 (d, C1), 109.8 and 113.7 (2 × s, C7 and C17), 128.0, 128.2, 128.4, 137.6 and 157.0 (2 × s and 5 × d, C3 and C Ph) p.p.m.. ESI-HRMS: *m/z* calculated for C_19_H_25_NO_6_ (MNa^+^) 386.1574, found 386.1579. C_19_H_25_NO_6_ (363.40 g mol^-1^): calculated C 62.80, H 6.93, N 3.85%; found C 63.08, H 7.45, N 3.89%.

### Refinement

H atoms were positioned geometrically and constrained to ride on the parent atom. The C—H bond distances are 0.98 Å for CH_3_, 0.99 Å for CH_2_ and 1.00 Å for CH. The *U*_iso_(H) values were set at 1.5*U*_eq_(C,*O*) for the CH_3_ groups and 1.2*U*_eq_(C) for all other H atoms. The value of the Flack parameter was not meaningful owing to the absence of significant anomalous scatterers, thus the data mere merged using MERG 4 in *SHELXL*. The absolute configuration was assigned by reference to the chiral starting material.

### Crystal data

**Table:**

C_19_H_25_NO_6_	*F*(000) = 388
*M**_r_* = 363.40	*D*_x_ = 1.249 Mg m^−^^3^
Monoclinic, *P*2_1_	Melting point: 403(1) K
Hall symbol: P 2yb	Mo *K*α radiation, λ = 0.71073 Å
*a* = 11.8012 (12) Å	Cell parameters from 1353 reflections
*b* = 6.0019 (5) Å	θ = 1.9–28.2°
*c* = 13.7021 (11) Å	µ = 0.09 mm^−^^1^
β = 95.122 (11)°	*T* = 293 K
*V* = 966.64 (15) Å^3^	Prism, colourless
*Z* = 2	0.30 × 0.05 × 0.04 mm

### Data collection

**Table:**

Stoe IPDS diffractometer	2023 reflections with *I* > 2σ(*I*)
Radiation source: fine-focus sealed tube	*R*_int_ = 0.104
graphite	θ_max_ = 28.2°, θ_min_ = 3.0°
Detector resolution: 6.0 pixels mm^-1^	*h* = −15→15
φ scans	*k* = −7→7
9449 measured reflections	*l* = −18→17
2558 independent reflections	

### Refinement

**Table:**

Refinement on *F*^2^	Primary atom site location: structure-invariant direct methods
Least-squares matrix: full	Secondary atom site location: difference Fourier map
*R*[*F*^2^ > 2σ(*F*^2^)] = 0.052	Hydrogen site location: inferred from neighbouring sites
*wR*(*F*^2^) = 0.131	H-atom parameters constrained
*S* = 0.99	*w* = 1/[σ^2^(*F*_o_^2^) + (0.0808*P*)^2^] where *P* = (*F*_o_^2^ + 2*F*_c_^2^)/3
2558 reflections	(Δ/σ)_max_ < 0.001
235 parameters	Δρ_max_ = 0.31 e Å^−^^3^
1 restraint	Δρ_min_ = −0.29 e Å^−^^3^

### Special details

**Table:**

Geometry. All e.s.d.'s (except the e.s.d. in the dihedral angle between two l.s. planes) are estimated using the full covariance matrix. The cell e.s.d.'s are taken into account individually in the estimation of e.s.d.'s in distances, angles and torsion angles; correlations between e.s.d.'s in cell parameters are only used when they are defined by crystal symmetry. An approximate (isotropic) treatment of cell e.s.d.'s is used for estimating e.s.d.'s involving l.s. planes.
Refinement. Refinement of *F*^2^ against ALL reflections. The weighted *R*-factor *wR* and goodness of fit *S* are based on *F*^2^, conventional *R*-factors *R* are based on *F*, with *F* set to zero for negative *F*^2^. The threshold expression of *F*^2^ > 2σ (*F*^2^) is used only for calculating *R*-factors(gt) *etc*. and is not relevant to the choice of reflections for refinement. *R*-factors based on *F*^2^ are statistically about twice as large as those based on *F*, and *R*-factors based on ALL data will be even larger.

### Fractional atomic coordinates and isotropic or equivalent isotropic displacement parameters (Å^2^)

**Table:**

	*x*	*y*	*z*	*U*_iso_*/*U*_eq_	
N1	−0.06053 (17)	0.2232 (4)	0.27810 (14)	0.0389 (4)	
O1	0.02900 (13)	0.5554 (3)	0.09073 (11)	0.0389 (4)	
O2	0.11981 (15)	0.8375 (3)	0.18283 (13)	0.0445 (4)	
O3	0.07981 (16)	0.6561 (4)	0.32044 (12)	0.0510 (5)	
O4	−0.06093 (18)	0.2884 (3)	0.37689 (13)	0.0477 (4)	
O5	−0.21517 (14)	0.5609 (3)	0.09506 (14)	0.0469 (4)	
O6	−0.34050 (19)	0.2785 (5)	0.0706 (3)	0.1008 (12)	
C1	0.01554 (19)	0.7319 (4)	0.15833 (17)	0.0371 (5)	
H1	−0.0423	0.8385	0.1321	0.044*	
C2	−0.01695 (19)	0.6276 (4)	0.25278 (17)	0.0363 (5)	
H2	−0.0841	0.7001	0.2758	0.044*	
C3	−0.04108 (17)	0.3891 (4)	0.22521 (16)	0.0325 (4)	
C4	−0.03742 (18)	0.3679 (4)	0.11580 (16)	0.0334 (4)	
H4	0.0012	0.2294	0.1004	0.040*	
C5	−0.15390 (19)	0.3778 (4)	0.05929 (18)	0.0384 (5)	
H5	−0.1455	0.3985	−0.0106	0.046*	
C6	−0.2320 (2)	0.1834 (5)	0.0731 (3)	0.0554 (7)	
H6A	−0.2283	0.0754	0.0208	0.067*	
H6B	−0.2119	0.1103	0.1354	0.067*	
C7	0.1722 (2)	0.7382 (5)	0.27004 (19)	0.0457 (6)	
C8	0.2295 (3)	0.9206 (6)	0.3320 (3)	0.0664 (9)	
H8A	0.2656	0.8576	0.3913	0.100*	
H8B	0.1738	1.0280	0.3479	0.100*	
H8C	0.2857	0.9922	0.2963	0.100*	
C9	0.2493 (3)	0.5495 (8)	0.2465 (3)	0.0773 (10)	
H9A	0.2837	0.4860	0.3063	0.116*	
H9B	0.3076	0.6048	0.2084	0.116*	
H9C	0.2058	0.4375	0.2099	0.116*	
C10	−0.0865 (2)	0.0973 (5)	0.43327 (18)	0.0494 (6)	
H10A	−0.0655	0.1285	0.5020	0.059*	
H10B	−0.0403	−0.0266	0.4146	0.059*	
C11	−0.2096 (2)	0.0296 (5)	0.42074 (18)	0.0452 (6)	
C12	−0.2392 (3)	−0.1738 (7)	0.4566 (4)	0.0787 (12)	
H12	−0.1830	−0.2674	0.4856	0.094*	
C13	−0.3520 (3)	−0.2411 (8)	0.4502 (4)	0.0966 (15)	
H13	−0.3711	−0.3789	0.4751	0.116*	
C14	−0.4337 (3)	−0.1078 (10)	0.4081 (4)	0.0921 (14)	
H14	−0.5095	−0.1515	0.4058	0.111*	
C15	−0.4060 (3)	0.0900 (11)	0.3690 (5)	0.114 (2)	
H15	−0.4623	0.1795	0.3376	0.137*	
C16	−0.2925 (3)	0.1590 (7)	0.3761 (3)	0.0835 (12)	
H16	−0.2738	0.2956	0.3498	0.100*	
C17	−0.3326 (2)	0.5127 (6)	0.0709 (3)	0.0613 (8)	
C18	−0.3984 (3)	0.6133 (11)	0.1487 (4)	0.1084 (18)	
H18A	−0.3931	0.7728	0.1458	0.163*	
H18B	−0.3674	0.5623	0.2119	0.163*	
H18C	−0.4767	0.5693	0.1380	0.163*	
C19	−0.3701 (3)	0.5986 (8)	−0.0303 (3)	0.0872 (12)	
H19A	−0.3649	0.7582	−0.0307	0.131*	
H19B	−0.4473	0.5545	−0.0481	0.131*	
H19C	−0.3218	0.5378	−0.0765	0.131*	

### Atomic displacement parameters (Å^2^)

**Table:**

	*U*^11^	*U*^22^	*U*^33^	*U*^12^	*U*^13^	*U*^23^
N1	0.0444 (10)	0.0412 (10)	0.0313 (9)	−0.0039 (8)	0.0040 (8)	−0.0003 (8)
O1	0.0417 (8)	0.0452 (9)	0.0306 (7)	−0.0071 (7)	0.0083 (6)	0.0006 (7)
O2	0.0443 (9)	0.0466 (10)	0.0421 (9)	−0.0176 (7)	0.0019 (7)	0.0041 (8)
O3	0.0495 (9)	0.0706 (12)	0.0317 (8)	−0.0259 (9)	−0.0027 (7)	0.0008 (8)
O4	0.0652 (11)	0.0476 (9)	0.0312 (8)	−0.0141 (8)	0.0090 (8)	−0.0014 (7)
O5	0.0343 (8)	0.0513 (10)	0.0536 (10)	0.0019 (8)	−0.0047 (7)	−0.0090 (9)
O6	0.0367 (10)	0.0663 (15)	0.199 (4)	−0.0063 (11)	0.0061 (15)	0.0190 (19)
C1	0.0360 (10)	0.0364 (10)	0.0383 (12)	−0.0056 (9)	0.0007 (9)	0.0028 (10)
C2	0.0349 (10)	0.0418 (11)	0.0323 (11)	−0.0041 (9)	0.0042 (8)	−0.0050 (9)
C3	0.0275 (9)	0.0375 (10)	0.0324 (11)	0.0007 (8)	0.0023 (8)	−0.0014 (9)
C4	0.0361 (10)	0.0327 (10)	0.0317 (10)	−0.0012 (8)	0.0050 (8)	−0.0017 (9)
C5	0.0398 (11)	0.0413 (11)	0.0335 (11)	−0.0010 (10)	−0.0002 (9)	−0.0070 (10)
C6	0.0411 (12)	0.0512 (15)	0.0721 (19)	−0.0080 (12)	−0.0050 (13)	−0.0109 (14)
C7	0.0400 (12)	0.0549 (14)	0.0416 (13)	−0.0101 (11)	0.0001 (10)	0.0039 (12)
C8	0.0580 (16)	0.077 (2)	0.0614 (19)	−0.0270 (16)	−0.0088 (15)	−0.0039 (16)
C9	0.0561 (17)	0.089 (3)	0.086 (2)	0.0134 (19)	−0.0004 (17)	0.001 (2)
C10	0.0557 (14)	0.0593 (16)	0.0330 (12)	−0.0121 (13)	0.0026 (10)	0.0076 (12)
C11	0.0511 (13)	0.0508 (14)	0.0350 (11)	−0.0015 (11)	0.0108 (10)	0.0022 (11)
C12	0.0552 (17)	0.073 (2)	0.108 (3)	−0.0078 (16)	0.0104 (18)	0.038 (2)
C13	0.061 (2)	0.089 (3)	0.141 (4)	−0.019 (2)	0.015 (2)	0.039 (3)
C14	0.0490 (17)	0.114 (3)	0.115 (4)	−0.004 (2)	0.016 (2)	0.008 (3)
C15	0.0470 (18)	0.126 (4)	0.169 (5)	0.019 (2)	0.009 (2)	0.051 (4)
C16	0.0572 (18)	0.075 (2)	0.119 (3)	0.0096 (17)	0.017 (2)	0.036 (2)
C17	0.0320 (11)	0.0644 (18)	0.086 (2)	0.0029 (12)	−0.0023 (12)	0.0015 (17)
C18	0.060 (2)	0.151 (5)	0.118 (4)	0.038 (3)	0.028 (2)	0.013 (4)
C19	0.0623 (18)	0.092 (3)	0.100 (3)	0.007 (2)	−0.0366 (19)	0.001 (2)

### Geometric parameters (Å, °)

**Table:**

N1—C3	1.265 (3)	C8—H8B	0.9600
N1—O4	1.410 (3)	C8—H8C	0.9600
O1—C1	1.425 (3)	C9—H9A	0.9600
O1—C4	1.431 (3)	C9—H9B	0.9600
O2—C1	1.398 (3)	C9—H9C	0.9600
O2—C7	1.426 (3)	C10—C11	1.504 (4)
O3—C2	1.416 (3)	C10—H10A	0.9700
O3—C7	1.429 (3)	C10—H10B	0.9700
O4—C10	1.430 (3)	C11—C16	1.352 (4)
O5—C17	1.426 (3)	C11—C12	1.373 (5)
O5—C5	1.426 (3)	C12—C13	1.386 (5)
O6—C6	1.400 (4)	C12—H12	0.9300
O6—C17	1.409 (4)	C13—C14	1.343 (7)
C1—C2	1.518 (3)	C13—H13	0.9300
C1—H1	0.9800	C14—C15	1.354 (7)
C2—C3	1.501 (3)	C14—H14	0.9300
C2—H2	0.9800	C15—C16	1.397 (6)
C3—C4	1.509 (3)	C15—H15	0.9300
C4—C5	1.517 (3)	C16—H16	0.9300
C4—H4	0.9800	C17—C18	1.501 (6)
C5—C6	1.509 (4)	C17—C19	1.508 (5)
C5—H5	0.9800	C18—H18A	0.9600
C6—H6A	0.9700	C18—H18B	0.9600
C6—H6B	0.9700	C18—H18C	0.9600
C7—C9	1.506 (5)	C19—H19A	0.9600
C7—C8	1.508 (4)	C19—H19B	0.9600
C8—H8A	0.9600	C19—H19C	0.9600
			
C3—N1—O4	110.36 (19)	H8A—C8—H8C	109.5
C1—O1—C4	109.48 (16)	H8B—C8—H8C	109.5
C1—O2—C7	108.55 (18)	C7—C9—H9A	109.5
C2—O3—C7	109.31 (17)	C7—C9—H9B	109.5
N1—O4—C10	108.36 (18)	H9A—C9—H9B	109.5
C17—O5—C5	106.0 (2)	C7—C9—H9C	109.5
C6—O6—C17	110.3 (2)	H9A—C9—H9C	109.5
O2—C1—O1	110.25 (18)	H9B—C9—H9C	109.5
O2—C1—C2	105.44 (18)	O4—C10—C11	113.8 (2)
O1—C1—C2	107.37 (19)	O4—C10—H10A	108.8
O2—C1—H1	111.2	C11—C10—H10A	108.8
O1—C1—H1	111.2	O4—C10—H10B	108.8
C2—C1—H1	111.2	C11—C10—H10B	108.8
O3—C2—C3	113.8 (2)	H10A—C10—H10B	107.7
O3—C2—C1	104.96 (18)	C16—C11—C12	118.4 (3)
C3—C2—C1	103.64 (19)	C16—C11—C10	123.3 (3)
O3—C2—H2	111.3	C12—C11—C10	118.3 (3)
C3—C2—H2	111.3	C11—C12—C13	120.6 (4)
C1—C2—H2	111.3	C11—C12—H12	119.7
N1—C3—C2	130.3 (2)	C13—C12—H12	119.7
N1—C3—C4	121.7 (2)	C14—C13—C12	120.2 (4)
C2—C3—C4	107.98 (19)	C14—C13—H13	119.9
O1—C4—C3	103.72 (17)	C12—C13—H13	119.9
O1—C4—C5	109.72 (18)	C13—C14—C15	120.2 (4)
C3—C4—C5	113.50 (18)	C13—C14—H14	119.9
O1—C4—H4	109.9	C15—C14—H14	119.9
C3—C4—H4	109.9	C14—C15—C16	119.8 (4)
C5—C4—H4	109.9	C14—C15—H15	120.1
O5—C5—C6	102.63 (19)	C16—C15—H15	120.1
O5—C5—C4	108.59 (18)	C11—C16—C15	120.8 (4)
C6—C5—C4	116.2 (2)	C11—C16—H16	119.6
O5—C5—H5	109.7	C15—C16—H16	119.6
C6—C5—H5	109.7	O6—C17—O5	105.4 (2)
C4—C5—H5	109.7	O6—C17—C18	111.4 (4)
O6—C6—C5	104.4 (2)	O5—C17—C18	107.9 (3)
O6—C6—H6A	110.9	O6—C17—C19	109.0 (4)
C5—C6—H6A	110.9	O5—C17—C19	110.1 (3)
O6—C6—H6B	110.9	C18—C17—C19	112.8 (3)
C5—C6—H6B	110.9	C17—C18—H18A	109.5
H6A—C6—H6B	108.9	C17—C18—H18B	109.5
O2—C7—O3	104.90 (19)	H18A—C18—H18B	109.5
O2—C7—C9	111.1 (3)	C17—C18—H18C	109.5
O3—C7—C9	110.2 (3)	H18A—C18—H18C	109.5
O2—C7—C8	107.9 (2)	H18B—C18—H18C	109.5
O3—C7—C8	107.6 (2)	C17—C19—H19A	109.5
C9—C7—C8	114.6 (3)	C17—C19—H19B	109.5
C7—C8—H8A	109.5	H19A—C19—H19B	109.5
C7—C8—H8B	109.5	C17—C19—H19C	109.5
H8A—C8—H8B	109.5	H19A—C19—H19C	109.5
C7—C8—H8C	109.5	H19B—C19—H19C	109.5
			
C3—N1—O4—C10	178.3 (2)	C3—C4—C5—C6	−68.1 (3)
C7—O2—C1—O1	−93.8 (2)	C17—O6—C6—C5	−8.4 (4)
C7—O2—C1—C2	21.8 (3)	O5—C5—C6—O6	25.7 (3)
C4—O1—C1—O2	139.51 (18)	C4—C5—C6—O6	144.0 (3)
C4—O1—C1—C2	25.1 (2)	C1—O2—C7—O3	−27.2 (3)
C7—O3—C2—C3	104.1 (2)	C1—O2—C7—C9	91.9 (3)
C7—O3—C2—C1	−8.6 (3)	C1—O2—C7—C8	−141.6 (2)
O2—C1—C2—O3	−8.0 (3)	C2—O3—C7—O2	21.7 (3)
O1—C1—C2—O3	109.5 (2)	C2—O3—C7—C9	−98.0 (3)
O2—C1—C2—C3	−127.70 (19)	C2—O3—C7—C8	136.4 (2)
O1—C1—C2—C3	−10.1 (2)	N1—O4—C10—C11	−74.9 (3)
O4—N1—C3—C2	1.8 (3)	O4—C10—C11—C16	−12.1 (4)
O4—N1—C3—C4	−179.14 (19)	O4—C10—C11—C12	168.0 (3)
O3—C2—C3—N1	58.5 (3)	C16—C11—C12—C13	−2.2 (7)
C1—C2—C3—N1	172.0 (2)	C10—C11—C12—C13	177.7 (4)
O3—C2—C3—C4	−120.6 (2)	C11—C12—C13—C14	0.4 (8)
C1—C2—C3—C4	−7.2 (2)	C12—C13—C14—C15	2.0 (9)
C1—O1—C4—C3	−28.9 (2)	C13—C14—C15—C16	−2.5 (9)
C1—O1—C4—C5	92.7 (2)	C12—C11—C16—C15	1.7 (7)
N1—C3—C4—O1	−157.54 (19)	C10—C11—C16—C15	−178.2 (5)
C2—C3—C4—O1	21.7 (2)	C14—C15—C16—C11	0.6 (9)
N1—C3—C4—C5	83.5 (3)	C6—O6—C17—O5	−12.4 (5)
C2—C3—C4—C5	−97.3 (2)	C6—O6—C17—C18	−129.1 (4)
C17—O5—C5—C6	−33.8 (3)	C6—O6—C17—C19	105.8 (4)
C17—O5—C5—C4	−157.3 (2)	C5—O5—C17—O6	29.4 (4)
O1—C4—C5—O5	−68.6 (2)	C5—O5—C17—C18	148.5 (3)
C3—C4—C5—O5	46.9 (3)	C5—O5—C17—C19	−88.0 (3)
O1—C4—C5—C6	176.4 (2)		

### Hydrogen-bond geometry (Å, °)

**Table:**

*D*—H···*A*	*D*—H	H···*A*	*D*···*A*	*D*—H···*A*
C5—H5···O2^i^	0.98	2.43	3.386 (3)	164
C10—H10A···O3^ii^	0.97	2.46	3.387 (3)	160

Symmetry codes: (i) −*x*, *y*−1/2, −*z*; (ii) −*x*, *y*−1/2, −*z*+1.
